# Balancing immune response: SHP1 controls neutrophil activation in inflamed lungs

**DOI:** 10.1172/JCI187056

**Published:** 2024-12-16

**Authors:** Laxman Ghimire, Hongbo R. Luo

**Affiliations:** Department of Pathology, Harvard Medical School and Brigham and Women’s Hospital, New Research Building, Boston, Massachusetts, USA.

## Abstract

Following respiratory infection or injury, neutrophil hyperactivation can damage surrounding lung tissue by releasing harmful compounds. In this issue of the *JCI*, Moussavi-Harami and colleagues identified tyrosine phosphatase SHP1 as a key negative regulator of neutrophil activation in acute respiratory distress syndrome (ARDS). Neutrophil-specific *Shp1* disruption leads to hyperinflammation, pulmonary hemorrhage, and increased mortality in both sterile and pathogen-induced acute lung injury (ALI). Large intravascular neutrophil clusters and excessive PAD4-independent neutrophil extracellular traps (NETs) were identified as key features of lung injury. Mechanistically, *Shp1* deficiency resulted in uncontrolled SYK kinase activation, driving chaotic neutrophil hyperactivation and inflammation.

## Effective host defense requires balanced neutrophil activation

Neutrophils are the first cells to be recruited to the site of infection, where they perform essential functions such as phagocytosis, degranulation, and the release of antimicrobial peptides and reactive oxygen species (ROS). They also form neutrophil extracellular traps (NETs) to kill or limit the spread of pathogens (1–3). However, hyperactivation or excessive recruitment of neutrophils can damage surrounding tissues by releasing harmful compounds such as oxidants, proteases, and DNA.

The importance of developing host-modulating therapies to counteract antimicrobial resistance is increasingly recognized. Immune defenses must be finely controlled during infection to balance pathogen clearance while limiting inflammation-induced tissue damage. An ideal antimicrobial treatment would enhance bactericidal activity while preventing neutrophil-driven inflammation and tissue damage. Disentangling the beneficial and harmful activities of neutrophils has proven exceptionally difficult, as they present with varied and often opposing functions that are influenced by the timing and location of the inflammatory response. To date, no single human drug either targets neutrophils or specifically leverages their unique biology for therapeutic purposes. To achieve this goal, there is a great need to further understand how various neutrophil functions are regulated, both positively and negatively, during infection and inflammation.

## SHP1 as a negative regulator of neutrophil activation

Src homology region 2 domain-containing phosphatase-1 (SHP1), a cytosolic tyrosine phosphatase, emerges as a crucial negative regulator that tempers neutrophil activity to prevent excessive inflammation (4). SHP1 is expressed predominantly in hematopoietic cells and, to a lesser extent, in endothelial and epithelial cells. It is recruited by inhibitory receptors through binding to immunoreceptor tyrosine-based inhibitory motifs (ITIM) and dephosphorylates proteins downstream of cytokine receptors, including GM-CSF1R, IL-3R, IL-4R, IL-13R, and interferon receptors, as well as TLR4 (5, 6). SHP1 also modulates neutrophil signaling by regulating the activation of ITAM-containing receptors, such as integrins and Fc receptors (5, 6). Upon engagement of the SH2 domains of SHP1 by tyrosine-phosphorylated ITIMs, SHP1 becomes activated, dephosphorylating key proteins, inhibiting ITAM-elicited signaling, and dampening downstream responses like ROS production, phagocytosis, and cytokine release (5–7). In patients, *SHP1* mutations are associated with neutrophilic dermatitis and chronic obstructive pulmonary disease (COPD). Global deficiency of *Shp1* in mouse models, known as motheaten mice, leads to autoimmunity, inflammatory dermatitis, pneumonitis, and death. Detailed cell-specific knockout experiments have established SHP1’s critical role in regulating myeloid lineage cells; cell-specific *Shp1* knockout in dendritic cells or neutrophils recapitulates aspects of the global loss of this protein in mice (5). Although SHP1 has been studied in the context of autoimmunity, its role in ALI has not been fully explored.

**Figure 1:**
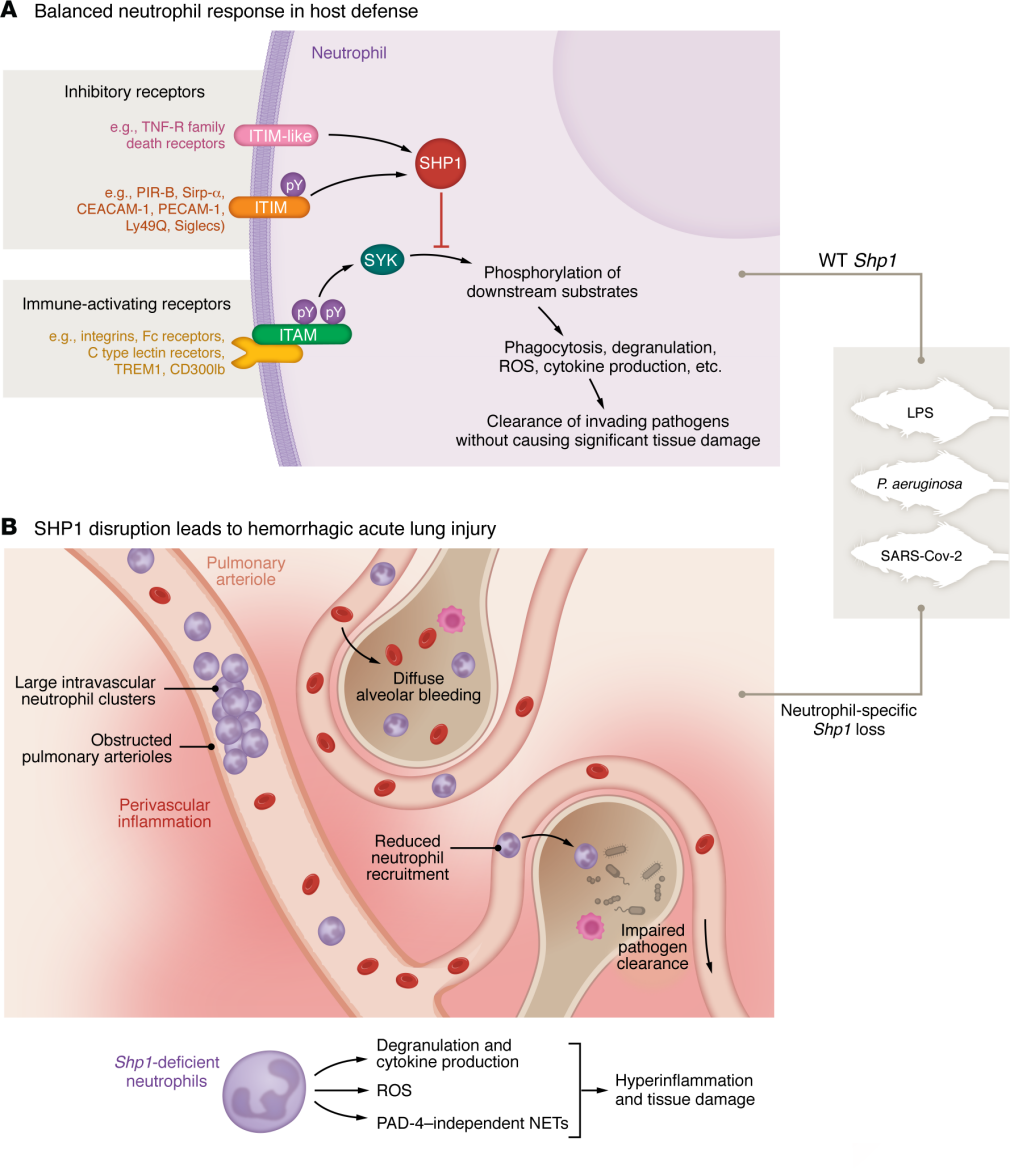
The SYK/SHP1 axis serves as a key regulator in neutrophil-mediated lung injury in ARDS. (**A**) In WT mice, SHP1 inhibits downstream neutrophil activation pathways initiated by SYK phosphorylation during acute lung injury caused by sterile agents (e.g., LPS), bacteria (e.g., *P*. *aeruginosa*), or viruses (e.g., SARS-CoV-2). As a result, the host effectively clears invading pathogens and/or resolves inflammation without causing substantial tissue damage. (**B**) However, acute lung injury in the absence of neutrophil Shp1 results in phosphorylated SYK that triggers excessive inflammatory cytokine production, degranulation, ROS release, and the formation of PAD-4–independent NETs, leading to perivascular inflammation and diffuse alveolar bleeding. SHP1 disruption–induced neutrophil hyperactivation also promotes the formation of large intravascular neutrophil clusters, leading to partial obstruction of the pulmonary arterioles, which, in turn, reduces neutrophil recruitment efficiency to infected alveolar spaces, thereby compromising their ability to eliminate bacteria. Together, these dysregulated neutrophil-driven events culminate in hemorrhagic and fatal acute lung injury.

In this issue of the *JCI*, Moussavi-Harami et al. (4) investigated the consequences of *Shp1* disruption in neutrophils and explored its potential as a therapeutic target for ALI. The authors used mouse models with specific *Shp1* deletion in neutrophils to assess its effects on lung injury in three acute lung inflammation models: sterile (lipopolysaccharide-induced; LPS), bacterial (*P*. *aeruginosa*), and viral (SARS-CoV-2). They observed excessive neutrophil recruitment, severe pulmonary hemorrhage, and increased NET formation in neutrophil-specific *Shp1*-knockout mice following intratracheal LPS challenge. The absence of *Shp1* led to the formation of large intravascular neutrophil clusters, contributing to vascular occlusion and exacerbating perivascular inflammation, likely impairing blood flow and worsening lung damage. Similar findings were reported using bacterial and viral models of ALI, suggesting that SHP1 is crucial in limiting neutrophil overactivation and preventing vascular injury during inflammation. Their findings highlight SHP1’s critical role in maintaining the balance between protective host defense responses and tissue-damaging hyperinflammation (Figure 1).

Interestingly, although overall NET formation was increased in *Shp1*-deficient mice, this NET formation occurred independently of Peptidyl Arginine Deiminase 4 (PAD4), which catalyzes histone citrullination, an important molecular trigger for NETosis and canonical NET formation (8, 9). Disruption of PAD4 did not rescue pulmonary hemorrhage and hyperinflammation associated with the loss of neutrophil *Shp1*. Thus, it appears that PAD4-independent NET formation is critical in these ALI models and that these NETs can destabilize the lung barrier, leading to hemorrhagic ALI. The underlying mechanism remains a direction for future research.

Another key observation from this study is that *Shp1* disruption did not simply enhance normal neutrophil bactericidal activity. In fact, the chaotic neutrophil hyperactivation led to impaired pathogen clearance. In the *P*. *aeruginosa* infection model, although neutrophils were hyperactive in terms of their phagocytosis and ROS production capabilities, the neutrophil-specific *Shp1* conditional-knockout mice showed impaired host defense against the pathogen, with increased colony-forming unit (CFU) levels. This impairment was likely due to the formation of abnormal large intravascular neutrophil clusters, which partially obstructed the pulmonary arterioles and reduced neutrophil recruitment efficiency to the infected alveolar space, thereby limiting the neutrophils’ ability to execute their bacteria-killing function. Supporting this notion, the authors observed that despite much higher CFU levels in neutrophil-specific *Shp1*-knockout mice, the number of neutrophils in the bronchoalveolar lavage fluid (BALF) was not substantially altered, indicating reduced neutrophil trafficking efficiency in the absence of *Shp1*.

## SHP1 maintains balanced neutrophil activation by suppressing SYK

Mechanistically, the neutrophil hyperactivity and pulmonary hemorrhage observed in *Shp1*-deficient mice are mediated through SYK kinase signaling, a key regulator of neutrophil adhesion, migration, degranulation, and ROS production (10, 11). SYK is a nonreceptor tyrosine kinase that plays a crucial role in immune signaling by transmitting signals from immune receptors. SHP1 functions as a downstream dephosphorylating regulator of proteins in these pathways (10, 12). Notably, genetic deletion of *Syk* in *Shp1*-deficient neutrophils ameliorated pulmonary hemorrhage and lung injury, indicating that the adverse effects of SHP1 loss are dependent on SYK kinase activity. This implicates the SYK/SHP1 axis as a major regulatory pathway in neutrophil-mediated lung injury, suggesting that targeting SYK kinase or modulating SHP1 activity could mitigate neutrophil-induced tissue damage in ARDS.

## SHP1 activation as a therapeutic strategy for ARDS

ARDS is a severe inflammatory condition of the lungs characterized by the sudden onset of respiratory failure, hypoxemia, and noncardiogenic pulmonary edema (13, 14). It is often accompanied by an overactive immune response, leading to excessive inflammation, tissue damage, and lung failure. Despite advances in supportive care, mortality rates in ARDS remain high (up to 40%), partly due to the complex nature of the disease, rapidly evolving pathophysiology, and patient heterogeneity (13, 15). Therefore, developing host-targeted therapies to treat ARDS is imperative. Neutrophil presence in ARDS patients potentially contributes to lung injury through the release of ROS, proteolytic enzymes, and NETs (16–18). Previous research into the immunobiology of ARDS has primarily focused on cytokine signaling receptors and immune-activating kinases. This report by Moussavi-Harami et al. (4) highlights that immune dysregulation associated with the loss of the inhibitory phosphatase SHP1 also exacerbates lung injury, as demonstrated in mice where the loss of neutrophil *Shp1* led to hyperinflammation and pulmonary hemorrhage in the context of sterile inflammation, as well as bacterial and viral infections.

A major translational aspect of the study involves the pharmacological activation of SHP1 to control neutrophil hyperinflammation. The small molecule SC43, a SHP1 activator, was shown to reduce neutrophil ROS production and alleviate inflammation in in vivo models of ALI. In LPS-challenged mice treated with SC43, there was a reduction in alveolar neutrophilia and NET formation, suggesting that pharmacological activation of SHP1 can dampen excessive immune responses. While SC43 reduced inflammation in WT mice, it was unable to reverse lung injury in *Shp1*-deficient mice, confirming that the beneficial effects of SC43 are mediated through SHP1 activation. This finding provides additional evidence that SHP1 plays a protective role in modulating neutrophil responses during ALI and that its activation could serve as a promising therapeutic approach for controlling neutrophil-mediated damage in ARDS.

## Concluding remarks

The study by Moussavi-Harami et al. provides compelling evidence that SHP1 plays a critical role in regulating neutrophil function and preventing hyperinflammation and pulmonary hemorrhage in ARDS. The loss of *Shp1* leads to unchecked neutrophil activation, resulting in severe pulmonary hemorrhage and lung injury. Importantly, the demonstrated efficacy of SHP1 activation in reducing inflammation highlights its therapeutic potential in ARDS. Moving forward, the clinical translation of SHP1-targeted therapies will require careful consideration of the balance between reducing inflammation and preserving essential immune functions. Further studies are needed to refine SHP1 activators, explore their effects in diverse models of lung injury, and assess their safety and efficacy in patients with ARDS.

Additionally, the degree of neutrophil activation can be controlled by SHP1, as well as by other previously identified negative regulators, such as phosphoinositide phosphatases (e.g., PTEN and SHIP) and certain inositol phosphates (e.g., InsP7 and Ins(1,3,4,5)P4) (19, 20). These regulators target distinct signaling pathways and may, therefore, govern different neutrophil functions. Host defense mechanisms against different pathogens vary and are often pathogen specific. A better understanding of the specific signaling pathways and cellular processes controlled, and not controlled, by each negative regulator could lead to more targeted, pathogen-specific personalized therapeutic approaches aimed at reducing neutrophil-mediated tissue damage without compromising the host defense against specific pathogens.

## Version 1 12/16/2024

Electronic publication
